# P081: Cefepime-tazobactam: a new antibiotic against ESBL producing enterobacteriaceae in cancer patients

**DOI:** 10.1186/2047-2994-2-S1-P81

**Published:** 2013-06-20

**Authors:** S Biswas, R Kelkar

**Affiliations:** 1 Microbiology, Tata Memorial Centre, Mumbai, India, Mumbai, India

## Introduction

India has very high rates of ESBL producing gram negative organisms and co-production of AmpC & OXA makes majority of antibiotics resistant, leaving carbapenems only reliable options. In recent times, E.coli and Klebsiella pneumoniae have also started showing resistance to carbapenems. Cefepime, a 4^th^ generation cephalosporin, is stable against AmpC & OXA, but it lacks activity against ESBL producing organisms. Clavulanate is a highly effective inhibitor of extended-spectrum beta-lactamases (ESBLs) in detection tests. A novel combination of Cefepime-tazobactam is expected to increase susceptibility of Enterobacteriaceae otherwise resistant to cefepime if used alone.The combination of Cefepime-tazobactam may effectively cover all three major resistance mechanisms (AmpC & OXA by cefepime, ESBLs by tazobactam).

## Objectives

This study was undertaken to estimate the prevalence of the ESBL producing gram negative bacilli and to evaluate the in vitro activity of the newer drug cefepime- tazobactam with piperacillin- tazobactum , cefoperazone sulbactam and carbapenems in a tertiary care cancer centre.

## Methods

This study was conducted between January 2012 to June 2012.All the samples were processed and identified as per routine microbiological methods. Antimicrobial susceptibility and ESBL confirmation was done following CLSI guidelines. Sensitivity of Cefepime-tazobactam was compared with cefepime, cefoperazone-sulbactam, piperacillin-tazobactam, imipenem, meropenem.

## Results

Of the269 isolates included in this study, there were 120 E.coli, 109 Klebsiella pneumoniae and 40 Enterobacter spp. 141 isolates were ESBL positive and 88 were ESBL negative.Carbapenems were the most sensitive followed by cefepime-tazobactam, cefepime, cefoperazone-sulbactam and pieracillin-tazobactam.

## Conclusion

ESBLs are novel beta-lactamases produced by a variety of gram-negative bacilli. Cefepime-tazobactam can be used for treatment of ESBL producing Enterobacteriaceae.This will help to reduce the usage of carbapenems in ESBL positive Enterobacteriaceae strains and prevent development of carbapenem resistance.This combination has shown good in-vivo response in many patients who were treated. Further detailed evaluation of this combination is required with in-vitro MIC studies and their correlation with clinical outcomes.

## Disclosure of interest

None declared

